# Olive Mill Wastewater Extract: In Vitro Genotoxicity/Antigenotoxicity Assessment on HepaRG Cells

**DOI:** 10.3390/ijerph21081050

**Published:** 2024-08-09

**Authors:** Tommaso Rondini, Raffaella Branciari, Edoardo Franceschini, Mattia Acito, Cristina Fatigoni, Rossana Roila, David Ranucci, Milena Villarini, Roberta Galarini, Massimo Moretti

**Affiliations:** 1 Department of Pharmaceutical Sciences, University of Perugia, Via del Giochetto, 06122 Perugia, Italy; tommaso.rondini@dottorandi.unipg.it (T.R.); edoardo.franceschini@unipg.it (E.F.); mattia.acito@gmail.com (M.A.); cristina.fatigoni@unipg.it (C.F.); milena.villarini@unipg.it (M.V.); 2 Department of Veterinary Medicine, University of Perugia, Via San Costanzo 4, 06126 Perugia, Italy; raffaella.branciari@unipg.it (R.B.); rossana.roila@unipg.it (R.R.); david.ranucci@unipg.it (D.R.); 3 Istituto Zooprofilattico Sperimentale dell’Umbria e delle Marche “Togo Rosati”, Via G. Salvemini 1, 06126 Perugia, Italy; r.galarini@izsum.it

**Keywords:** olive by-products, phenolic compounds, comet assay, viability assay

## Abstract

Olive mill wastewater (OMWW), with its high level of phenolic compounds, simultaneously represents a serious environmental challenge and a great resource with potential nutraceutical activities. To increase the knowledge of OMWW’s biological effects, with an aim to developing a food supplement, we performed a chemical characterisation of the extract using the Liquid Chromatography–Quadrupole Time-of-flight spectrometry (LC–QTOF) and an in vitro genotoxicity/antigenotoxicity assessment on HepaRG ™ cells. Chemical analysis revealed that the most abundant phenolic compound was hydroxytyrosol. Biological tests showed that the extract was not cytotoxic at the lowest tested concentrations (from 0.25 to 2.5 mg/mL), unlike the highest concentrations (from 5 to 20 mg/mL). Regarding genotoxic activity, when tested at non-cytotoxic concentrations, the extract did not display any effect. Additionally, the lowest tested OMWW concentrations showed antigenotoxic activity (J-shaped dose–response effect) against a known mutagenic substance, reducing the extent of DNA damage in the co-exposure treatment. The antigenotoxic effect was also obtained in the post-exposure procedure, although only at the extract concentrations of 0.015625 and 0.03125 mg/mL. This behaviour was not confirmed in the pre-exposure protocol. In conclusion, the present study established a maximum non-toxic OMWW extract dose for the HepaRG cell model, smoothing the path for future research.

## 1. Introduction

Olive mill wastewaters (OMWW) are the main liquid effluents generated by the olive oil production industry.

Mediterranean countries produce more than 2.8 million metric tonnes of olive oil per year with about 71% of the worldwide production coming from Europe; Spain (44.5% of global production) and Italy (10.1% of global production) are the top producers. Production worldwide has increased by ca. 48% over the last decade [1].

The composition of OMWW, which displays many organic compounds, particularly, phenolic compounds [2], causes serious economic and technical barriers to efficient effluent treatment and disposal [3]. Furthermore, the disposal of OMWW might impact the soil’s physical and chemical properties and plant seed germination due to the phytotoxic activity of phenolics [4,5].

To reduce this significant environmental burden, the possible reuse of olive oil mill by-product extracts in several fields has been evaluated over time.

Most polyphenols present in olives are lost in wastes (olive mill wastewater and pomace) during oil extraction [6]. Consequently, several methods have been evaluated to recover these compounds [7], which are associated with numerous positive biological activities, turning olive oil by-products into an inexpensive source of natural antioxidants.

Polyphenol extract from OMWW has displayed antimicrobial activities by retarding the growth of *Pseudomonas fluorescens* and *Enterobacteriaceae* isolated from “mozzarella” and “fior di latte” cheese, thus improving their shelf life [8,9,10]. 

Appropriately treated, OMWW was also used as a dietary supplement in dairy sheep to enhance cheese quality with respect to the antioxidant status and oxidative stability without altering its quality and chemical parameters [11].

Recently, the use of recovered phenolic compounds from OMWW has been assessed for the green synthesis of magnesium sulphide nanoparticles, which have been shown to promote the germination of old pea seeds and soil bacteria [12]. 

The human health benefits of substances derived from olives are widely known [13]. These benefits are mainly related to the antioxidant effect of phenolic compounds [14], which are associated with reducing risk factors for coronary heart disease, preventing different types of cancers, and modulating immune and inflammatory responses. Consequently, virgin olive oil is considered an example of a functional food [15]. 

At least 30 phenolic compounds have been detected in OMWW [5]. Generally, these compounds present in olive pulp (and sometimes in the stones) are often more soluble in the water phase than in oil, showing remarkable concentrations (0.5 to 25 g/L) in OMWW [16].

Given the significant impact of olive compounds on human health [5], this study aimed to conduct both chemical characterisation and an in vitro genotoxicity/antigenotoxicity assessment of OMWW extract, obtained from the Department of Veterinary Medicine of the University of Perugia (Perugia, Italy), with the goal of developing a food supplement. 

Since most market withdrawals of pharmaceutical products are related to hepatotoxicity [17,18,19,20], it is essential to carry out an in vitro hepatic toxicity assessment from the early phases of investigation. Food supplements are generally administered per os, absorbed in the gastrointestinal tract, and reach the liver, often undergoing first-pass metabolism in this organ.

In this study, we have evaluated the cytotoxicity and genotoxicity in HepaRG^TM^, a pre-clinical hepatic model.

## 2. Materials and Methods

### 2.1. Chemicals and Reagents

William’s E medium, foetal bovine serum (FBS), trypsin-EDTA, L-glutamine, antibiotics (penicillin and streptomycin), and insulin were bought from Euroclone SpA (Milan, Italy). Hydrochloric acid (HCl), dimethyl sulfoxide (DMSO), ethanol, ethylenediaminetetracetic acid disodium (Na_2_EDTA) and tetrasodium (Na_4_EDTA) salt, sodium chloride (NaCl), and sodium hydroxide (NaOH) were obtained from Carlo Erba Reagenti Srl (Milan, Italy). Dulbecco’s phosphate-buffered saline, pH 7.4 (PBS), ethidium bromide, low- and normal-melting-point agarose (LMPA and NMPA, respectively), 4-nitroquinoline N-oxide (4NQO), tris(hydroxymethyl)aminomethane (Tris base), Triton X-100, and GlutaMax were obtained from Merck Life Science (Merck KGaA, Darmstadt, Germany). Acridine orange (AO), 4′,6′-diamidino-2-phenylindole (DAPI), and NC-Slide A8 were bought from ChemoMetec A/S (Allerød, Denmark). Conventional microscope slides and coverslips were purchased from Knittel-Glaser GmbH (Braunschweig, Germany). Distilled water was used throughout the experiments.

### 2.2. Characteristics of Polyphenols Extracts and Determination of the Composition by LC–QTOF

The analysed polyphenol extracts were extracted from olive mill wastewater provided by Stymon Natural Products P.C., Patras, Greece (www.stymon.com). The product is derived from the OMWW of the olive (*Olea europea* L.), specifically a Koroneiki cultivar. 

Since polyphenols are easily degradable by environmental and processing conditions (oxygen, water, light, etc.) that restrict their efficiency and stability, the extracts were encapsulated through freeze-drying after being combined with a maltodextrin carrier (1:1 dw). Maltodextrin, as amorphous carbohydrate microstructure matrices, has proven to be the most suitable method for drying delicate and thermosensitive biomaterials, minimising the impact of thermal degradation and chemical alteration, and protecting them from undesirable physical phenomena [21,22,23]. 

The concentrations of polyphenol extracts were determined by liquid chromatography–quadrupole time-of-flight spectrometry (LC–QTOF) as reported by Roila et al. [24]. The platform consisted of an Exion LC™ coupled to a 6600+ TripleTOF™ (AB Sciex, Foster, CA, USA) equipped with an electrospray ionisation source operating in negative ionisation mode (ESI-). Chromatographic separation was carried out using an Acquity BEH C18 column (150 mm × 2.1 mm, 1.7 μm, Waters, Milford, MA, USA). The mobile phases were methanol/ACN 90/10 *v*/*v*% (B) and water with 0.025% acetic acid (A). The gradient began with 0% of B for 1 min; B increased to 20% after an additional 10 min, followed by B increasing to 50% after a further 4 min, and finally reaching 100% of B after an additional 3 min. After holding the system at 100% of B for 5 min, the percentage of B returned to 0% over 1 min. The system was re-equilibrated for six minutes. The autosampler was maintained at 25 °C, whereas the column was set at 40 °C. The injection volume was 10 µL, and the flow rate was 0.25 mL/min. The curtain gas (40 psi) was nitrogen, while the GS1 (55 psi) and GS2 (55 psi) were compressed air. The interface source temperature was set at 450 °C, and the spray voltage was set at −4.5 kV.

### 2.3. Cell Culture

The experimental design included tests on undifferentiated HepaRG liver cells. These cells originated from a tumour of a female patient suffering from chronic hepatitis C infection and hepatocellular carcinoma [25]. Compared with other liver experimental models, they are most similar to primary human hepatocytes and liver tissues [26], retaining many attributes and primary human functions of hepatocytes, such as morphology and gene expression (CYPs, transporters of Phase II metabolism) [27]. Therefore, HepaRG cells can be a useful model for in vitro studies on drug metabolism and toxicity [28]. 

HepaRG cells (cryopreserved, catalogue # HPRGC10) were purchased from Life Technologies Italia (Monza, Italy). Cells were cultured in 75 cm^2^ flasks with the growth medium composed of William’s E medium (Invitrogen) supplemented with inactivated foetal bovine serum (10%), insulin (5 µg/mL), hydrocortisone hemisuccinate (50 µM), GlutaMax (1%), and antibiotics (penicillin 100 U/mL, streptomycin 0.1 mg/mL), at 37 °C in a humidified atmosphere with 5% CO_2_. Trypsin treatment detached cells from flasks and was passaged for in vitro treatments.

Analyses were performed on HepaRG from passages 9 to 14.

### 2.4. OMWW Extract Preparation

According to the Organisation for Economic Co-operation and Development (OECD) document No. 129 [29], appropriate tests were carried out, showing the solubility of the OMWW dry extract directly in the cell growth medium. Thus, a stock solution at the concentration of 20 mg/mL was prepared.

### 2.5. Cytotoxicity Testing—AO/DAPI Double Staining 

In line with the Organization for Economic Co-operation and Development (OECD) guidelines [30], which recommend starting from a concentration of at least 5 mg/mL when the tested extract composition is unknown, the potential cytotoxicity of the study compound was evaluated by testing different concentrations (i.e., 20, 15, 10, 7.5, 5, 2.5, 1.25, 1, 0.5, and 0.25 mg/mL), starting from the stock solution. For this test, concentrations up to 20 mg/mL were considered in order to broaden the range of the potential cytotoxic activity of the olive by-product extract.

The cell line was seeded overnight at a density of 2.65 × 10^5^ cells/cm^2^ in a 12-well plate (Corning Inc., Corning, NY, USA) in 1 mL volume, achieving a semiconfluent monolayer. Afterward, they were treated with 1% Triton X-100 (positive control) and OMWW extract at different concentrations for 4 h. 

Appropriate negative controls (untreated cells) were also included.

The number of total and viable cells were rated by staining cell populations with Acridine Orange (AO) and 4′,6-diamidino-2-phenylindole (DAPI) fluorophores, as described elsewhere [31,32]. Briefly, AO, a membrane-permeable dye, can bind to nucleic acids, staining every single cell of the sample. Alternatively, DAPI, which is not membrane-permeable, can only stain non-viable cells with a damaged cell membrane.

After the treatment, the supernatant (containing floating cells) and adherent cells were collected in 15 mL centrifuge tubes and centrifuged at 500× *g* for 5 min. Pellets were rinsed with 1 mL PBS, centrifuged at 500× *g* for 5 min, and suspended in 1 mL PBS. Cell suspensions were transferred to 2 mL test tubes and vortexed for a few seconds.

Finally, samples were treated with the AO/DAPI dye solution and loaded onto NC-Slide A8. The slide was then inserted in the fluorescence-based image cytometer NucleoCounter^®^ NC-3000™ (Chemometec, Allerød, Denmark), which displayed cell concentration and viability. 

### 2.6. Genotoxicity/Antigenotoxicity Testing

Both genotoxicity and antigenotoxicity assays were performed according to previous procedures [32,33,34,35] with minor modifications. HepaRG cells were seeded at approximately 2.65 × 10^5^ cells/well in 12-well plates (Corning Inc., Corning, NY, USA) before treatment with OMWW extracts.

Each test was set up in triplicate, and all steps were carried out under yellow light to prevent additional DNA damage.

To avoid conditions that would lead to false-positive results arising from DNA damage correlated with cytotoxicity [36], non-cytotoxic concentrations of the OMWW extract (i.e., 2.5, 1.25, 1, 0.5 and 0.25 mg/mL) were tested in the comet assay.

For the antigenotoxicity assay, after a preliminary test, another test was performed by adding some lower scalar concentrations (from 0.125 to 0.015625 mg/mL) to verify a potential hormetic tendency. 

#### 2.6.1. Genotoxicity Treatment

The day after the seeding, the growth medium was removed, and cells were exposed to different OMWW scalar concentrations (from 2.5 to 0.25 mg/mL) for 4 h. 

A positive control with model mutagen 4NQO (2 µM) and two negative controls, one with only cell medium and one with DMSO (1%) diluted in the medium, were included.

#### 2.6.2. Antigenotoxicity Treatments

An appropriate positive control was included for each antigenotoxicity test using 4NQO, a known mutagen and carcinogen widely used in the comet assay [37]. 

##### Co-Exposure Treatment 

In this case, after removing the culture medium, HepaRG cells were treated according to the following scheme:

- Challenge cultures: William’s E medium containing serial dilutions of OMWW extract (i.e., 0.015625, 0.03125, 0.0625, 0.125, 0.25, 0.50, 1.00, 1.25, and 2.50 mg/mL) plus 2 µM 4NQO.
- Positive control (known mutagen cultures): Fresh complete William’s E medium plus 2 µM 4NQO.
- Solvent control: Fresh complete William’s E medium plus 1% DMSO.
- Negative control: Untreated cells.

At the end of this treatment, the cells were processed for the comet assay as described in Section 2.7.

##### Post-Exposure Treatment 

After a 24 h culture, the culture medium was replaced by fresh complete William’s E medium containing 2 µM 4NQO, and the cells were further incubated for 4 h. 

After the culture medium was removed, the cells were washed in PBS. Fresh complete William’s E medium with serial dilution of the OMWW extract (see co-exposure), was added, and they were further incubated for 4 h. 

Appropriate positive control (i.e., 2 µM 4NQO), solvent control (i.e., 1% DMSO), and negative control (i.e., untreated cells) were included in each experimental set.

At the end of this treatment, the cells were processed for the comet assay as described in Section 2.7.

##### Pre-Exposure Treatment

After a 24 h culture, the culture medium was replaced by fresh complete William’s E medium containing the test concentrations of the OMWW extract (see “Co-Exposure Treatment”). The cells were then incubated further for 4 h. 

Subsequently, the culture medium containing the OMWW extract was removed, the cells washed in PBS, and fresh complete William’s E medium was added. They were then incubated further for 4 h. 

Appropriate positive control (i.e., 2 µM 4NQO), solvent control (i.e., 1% DMSO), and negative control (i.e., untreated cells) were included in each experimental set. 

At the end of this treatment, the cells were processed for the comet assay as described in Section 2.7.

### 2.7. Alkaline Single-Cell Microgel Electrophoresis (Comet) Assay

After the treatments, cells were detached by trypsin and subjected to centrifugation (70× *g*, 8 min, 4 °C). The pellets were resuspended in 300 µL of 0.7% LMPA (in Ca^2+^/Mg^2+^-free DPBS (*w/v*)) and layered onto microscope slides pre-coated with 1% NMPA in Ca^2+^/Mg^2+^-free DPBS (*w/v*). Coverslips were placed onto the samples and removed after allowing the agarose to solidify for 10 min at 4 °C, followed by another top layer of 75 µL of 0.7% LMPA. The slides were then dipped overnight at 4 °C in a lysing solution, composed of 2.5 M NaCl, 100 mM Na_2_EDTA, and 10 mM Tris-HCl, pH 10, containing 1% Triton X-100. 

To allow the expression of alkali-labile damage and the unwinding of DNA, the slides were maintained for 20 min in an alkaline buffer solution (pH > 13) made up of 10 mM Na_4_EDTA and 300 mM NaOH. Electrophoresis was performed with a horizontal box (HE99; Hoefer Scientific, Holliston, MA, USA) at an electric field strength of 1 V/cm, with current at 300 mA (Power Supply E411; Consort, Turnhout, Belgium) in an ice bath for 20 min. After electrophoresis, the microgels were neutralised with 0.4 M Tris-HCl buffer, pH 7.5.

The slides were finally fixed in ethanol for 10 min and stored in slide boxes at room temperature. 

To evaluate DNA damage, the slides were stained with ethidium bromide (50 µL, 20 µg/mL) and examined using an Olympus BΧ41 (Japan) fluorescence microscope equipped with a high-sensitivity charge-coupled device (CCD) camera connected to a computerised image analysis system (“Comet Assay III”, Perspective Instruments Ltd., Suffolk, UK). One hundred randomly selected cells (50 cells/replicate slides) were analysed, with tail intensity (%) being chosen as the damage parameter. The median of the scored comets for each slide was used to calculate the group means [38].

### 2.8. Statistical Analysis

Cytotoxicity and genotoxicity/antigenotoxicity data are expressed as the mean ± standard error of the mean (SEM) of triplicate tests. Statistical significance was assessed using one-way ANOVA followed by Dunnett’s post hoc test at a significance level of *p* < 0.05.

## 3. Results

### 3.1. Characteristics of Polyphenols Extract and Determination of the Composition by LC–QTOF

**Figure 1:**
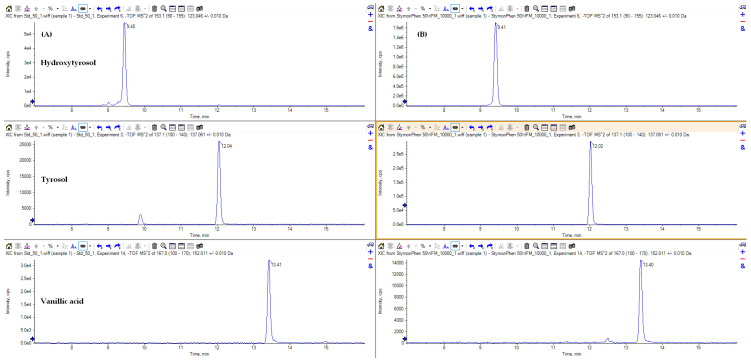
Extract ion chromatograms of the three most abundant polyphenols: hydroxytyrosol, tyrosol, and vanillic acid. (**A**) Standard solution at 50 ng/mL; (**B**) extract (10,000-fold dilution).

The specific content of the phenolic compounds was 14.3 for hydroxytyrosol, 3.42 for tyrosol, and 0.32 mg/g for vanillic acid, respectively (Figure 1).

### 3.2. Cytotoxicity Testing—AO/DAPI Double Staining

The test was performed by treating cells with different concentrations (specified in the experimental section) of OMWW extract. 

**Figure 2:**
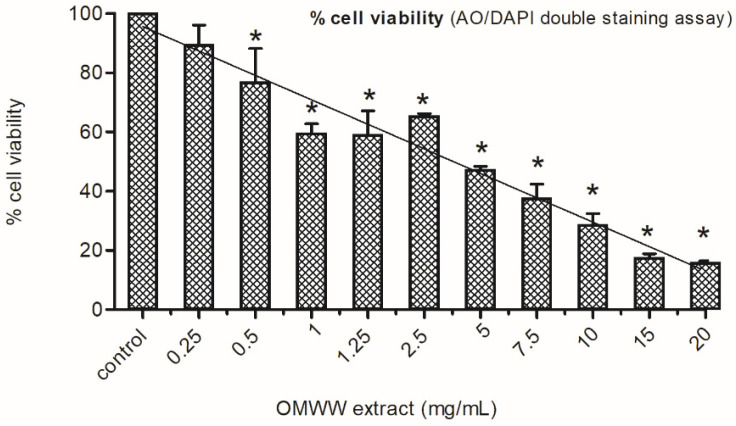
Effect of OMWW extract on cell viability in HepaRG cells after 4 h exposure. Cytotoxic effects were assessed with a AO/DAPI double staining test. Results are summarised as mean ± SEM of three independent experiments. Control = untreated cells. Results are expressed as normalised viability compared with the control (=100% of viability); * *p* < 0.05 vs. control.

A statistically significant (*p* < 0.05), concentration-dependent effect was observed (Figure 2). 

According to OECD guidelines [30], which established <55 ± 5% as a cytotoxic threshold value, the highest non-cytotoxic OMWW concentration was 2.5 mg/mL (65.5 ± 0.7% viability). Furthermore, the percentages of cell viability, ranging from 37.6 ± 4.8% to 15.9 ± 0.5%, highlight a clear cytotoxic effect of the extract from the 7.5 mg/mL concentration onward.

### 3.3. Comet Assay

#### 3.3.1. Genotoxicity Testing

**Figure 3:**
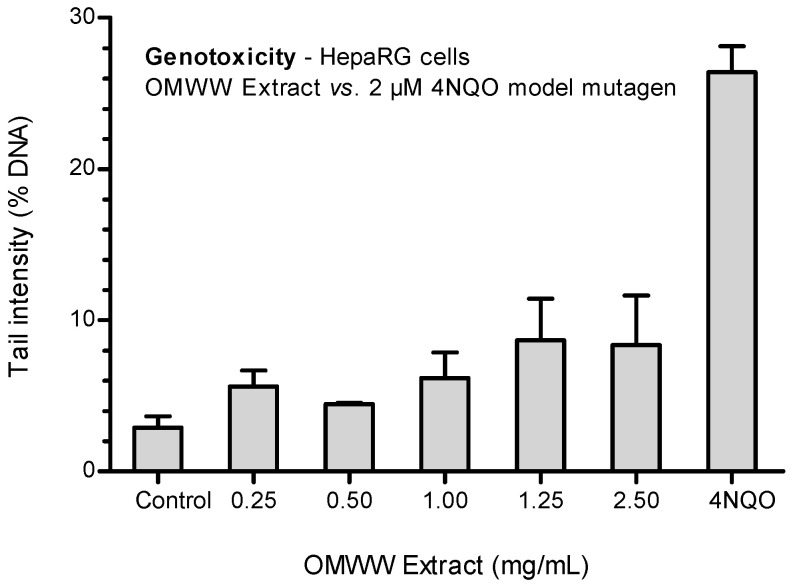
Genotoxic effects of OMWW extract in HepaRG cells after 4 h of exposure. Each result is expressed as the mean ± SEM of three independent experiments. Control (untreated cells) = 2.89 ± 0.76%; Positive control (2 μM 4NQO).

The genotoxicity testing results are displayed in Figure 3.

An increased concentration-dependent tendency was observed, with higher levels of DNA damage (expressed in tail intensity %) at the highest concentrations (8.7% and 8.6% for 2.5 mg/mL and 1.25 mg/mL OMWW extract concentration, respectively), although without statistically significant evidence (*p* = 0.373). 

#### 3.3.2. Antigenotoxicity Testing

##### Co-Exposure

**Figure 4:**
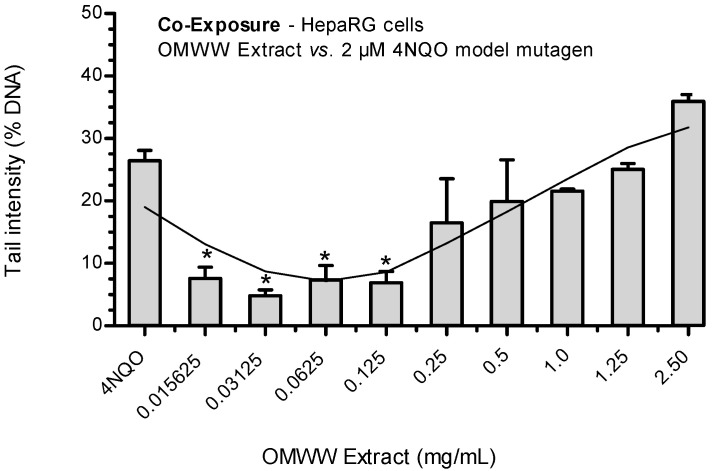
Antigenotoxic effects of OMWW extract on 4NQO-induced DNA damage in HepaRG cells (co-exposure protocol) and genotoxic inhibition ratio toward model mutagen 4NQO (2 μM). Each result is expressed as the mean ± SEM of three independent experiments. Control (untreated cells) = 3.45 ± 1.48%; positive control (2 μM 4NQO); * *p* < 0.05 vs. 2 μM 4NQO.

Simultaneous treatment of cells with the mutagen 4NQO (2 µM) and different scalar concentrations of OMMW extract showed a statistically significant (*p* < 0.05) J-shaped dose–response curve of residual DNA (Figure 4).

The OMWW extract displayed a clear antigenotoxic effect at concentrations of 0.015625, 0.03125, 0.625, and 0.125 mg/mL, with very similar values of tail intensity (7.6%, 4.8%, 7.4%, and 6.9%, respectively).

This effect decreased as the tested concentrations of the extract increased, reaching the highest extent of DNA damage at 2.5 mg/mL. 

##### Pre-Exposure

In this case, the first 4 h treatment was done using different scalar concentrations of OMWW extract, followed by treatment with the known mutagen compound 4NQO (2 µM).

**Figure 5:**
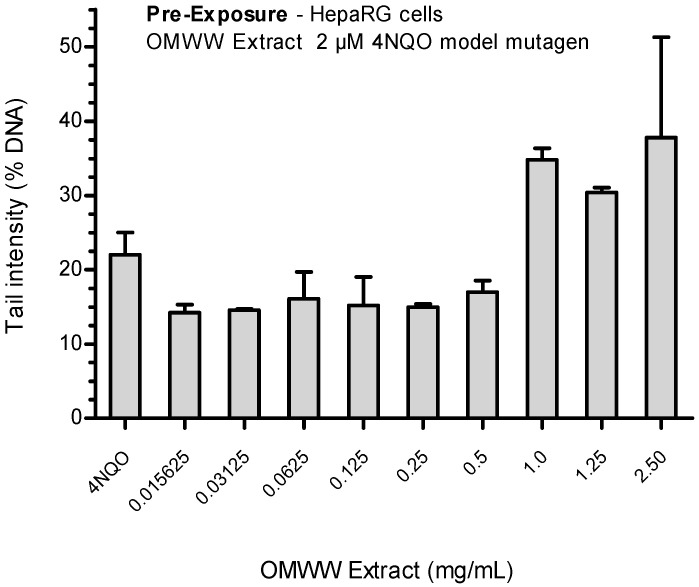
Antigenotoxic effects of OMWW extract on 4NQO-induced DNA damage in HepaRG cells: pre-exposure protocol. Each result is expressed as the mean ± SEM of three independent experiments. Control (untreated cells) = 3.91 ± 0.75%; positive control (2 µM 4NQO).

An increased concentrations-dependent trend was observed in the extent of DNA damage (Figure 5), though without statistical significance (*p* > 0.05). 

At the three highest OMWW extract concentrations (1.0, 1.25 and 2.50 mg/mL), a consistent increase in DNA damage was evident, with tail intensity values of 34.80%, 30.40%, and 37.79%, respectively, which are almost double compared to the ones obtained for the lowest concentrations.

##### Post-Exposure

**Figure 6:**
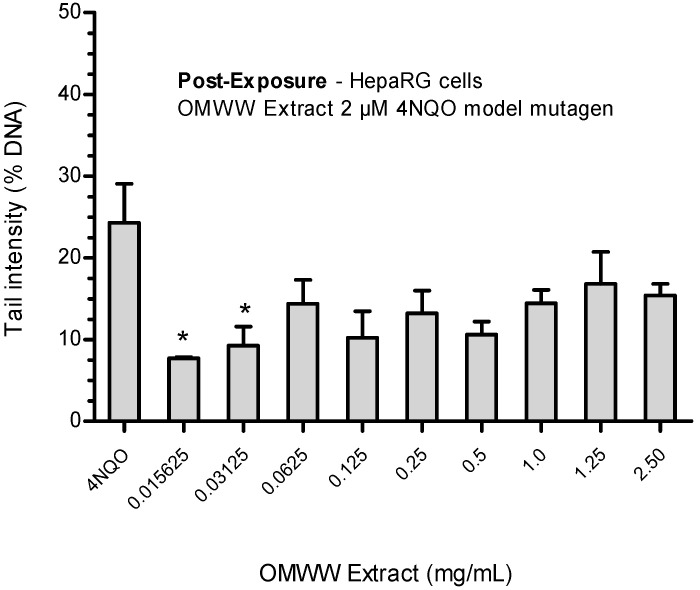
Antigenotoxic effects of OMWW extract on 4NQO-induced DNA damage in HepaRG cells: post-exposure protocol. Each result is expressed as the mean ± standard error of the mean (SEM) of three independent experiments. Control (untreated cells) = 3.95 ± 0.75%; positive control (2 µM 4NQO); * *p* < 0.05 vs. 2 µM 4NQO.

After exposing cells previously to the mutagen (4NQO 2 µM) and then the OMWW extract, antigenotoxic activity (*p* < 0.05) was present only at the lowest tested concentrations (0.015625 and 0.03125 mg/mL) (Figure 6). 

Subsequently, the addition of increasing concentrations showed an almost constant level of tail intensity, in the range of 10–16%. 

## 4. Discussion

OMWW, a stable emulsion of vegetation water from olives [39], represents an environmental challenge. The large volume produced and its chemical composition make its disposal a relevant issue because it can compromise the balance of the ecological system [5]. For these reasons, the possibility of its reuse has been considered over time [40].

In this study, a chemical investigation and in vitro genotoxicity/antigenotoxicity assessments of HepaRG cells, an undifferentiated liver cell line close to primary human hepatocytes and liver tissues, were performed in order to evaluate the biological effect of the OMWW extract with the aim of eventually developing potential food supplements.

A determination of the main phenolic compounds of our extract was done using LC–QTOF. Consistent with other studies [41,42], hydroxytyrosol was the most abundantly detected phenolic substance.

Subsequently, the in vitro cytotoxic and genotoxic/antigenotoxic effects of the extract at different concentrations were determined using the AO/DAPI double staining cell count and viability assay as well as the alkaline comet assay (alkaline unwinding/alkaline electrophoresis, pH > 13).

Focusing on other by-products of the olives, an in vitro study on HepG2 liver cells evaluated the cytotoxic effects of olive leaf extract [43]. However, to our knowledge, the present study is the first experiment performed with OMWW extract on a hepatic cell line. Most studies have focused on the in vitro anticancer activity of OMWW on different cell lines, including breast cancer [44], bladder cancer [45], lung cancer [46], and prostate cancer [47].

Concerning the lowest concentrations tested in our work (from 2.5 to 0.25 mg/mL), which are comparable to those evaluated by other studies on different cell lines [48,49], treatment of the cells with OMWW extract did not show any direct cytotoxicity. On the contrary, the significant reduction in cell viability was observed only at the highest extract concentrations (from 5 to 20 mg/mL). This cytotoxic activity is not suggested by other studies, which, however, tested far lower concentrations of OMWW extract [49].

Several works focused on different fractions of OMWW, such as phenolic ones [50,51,52]. In particular, Silvan et al. [53] demonstrated the cytotoxic activity of an OMWW phenolic fraction on two intestinal cell lines (HT-29 and Caco-2). In this case, the concentrations tested were almost equal to the lowest ones used in our study (from 2.5 to 0.25 mg/mL). However, these results must be compared cautiously since the goal of the present study was not to evaluate the effect of a fraction of the olive mill wastewater but of the whole phyto-complex.

The genotoxicity test did not display any significant effect, as also reported by Mitakou and co-workers on Jukart cells [48].

For the antigenotoxicity test, we assessed OMWW extract for its protective properties against the known mutagen 4NQO, which is widely used in genotoxicity studies. 4NQO is an oxidative mutagen that can bind to DNA, inducing its strand breakage [54]. These breaks represent a wide spectrum of DNA lesions (e.g., DNA adducts, apurinic/apyrimidinic sites, etc.) detectable by the comet assay. Simultaneously exposing HepaRG cells to the extract and the 4NQO revealed a J-shaped hormetic tendency. The hormetic response can be defined as an adaptative, non-monomatic, biphasic dose–response relationship [55], which has been observed in several plant-based products and/or compounds such as propolis and red wine resveratrol [34,35,56]. Our results showed clear antigenotoxic activity of the OMWW extract at the lowest concentrations, reaching the highest activity against the known mutagen at 0.03125 mg/mL (8.6 ± 1.63% tail intensity).

To the best of our knowledge, the hormetic effect of this product has never been depicted in any study before. An in vitro study on Jukart cells [48] highlighted a concentration-dependent effect, demonstrating interesting DNA damage-reducing properties of OMWW extract pre-incubated with H_2_O_2_. This study also compared the effects of the extract with other olive products/by-products (i.e., leaves, fruits, and oil). Protective and genotoxic agents were observed in olive leaves, fruits, and oil extracts, while only protective compounds were found in the OMWW extract. Indeed, OMWW did not show the genotoxic activity at any concentration. These findings are in line with our study, where a similar effect was observed at comparable concentrations.

The mitigating activity toward toxic effects caused by certain types of substances was also shown on mammalian myocytes (H9C2) co-exposed to OMWW and some chemotherapy drugs (i.e., 5-fluorouracil) [44].

Moreover, treating cells first with the mutagen (4NQO) and then with the OMWW extract (post-exposure procedure) displayed antigenotoxic activity solely at the lowest tested concentrations (0.015626 and 0.03125 mg/mL). 

On the contrary, no statistically significant effects were observed in the pre-exposure treatment. 

The results we observed may be at least partially correlated to the high presence of hydroxytyrosol in the extract. According to other studies on hepatic cancer cell lines (HepG2) [57,58], this molecule has displayed a cytotoxic effect by itself at concentrations lower than those found in our tested extract. However, it is important to highlight that the extract by-product contains several molecules that can interact with hydroxytyrosol, resulting in the observed activity.

In summary, our results demonstrated that, when considered as phyto-complex, the bioactive molecules within the extract contribute to interesting biological properties that deserve deeper investigation.

As an in vitro study, the present work can only be considered a preliminary research approach. Future research directions might include further tests on other cell lines, such as intestinal ones, and in vivo studies, which would be useful and necessary to complete and corroborate the present study, for the development of potential food supplements. 

## 5. Conclusions

The present study expanded the knowledge of this olive oil by-product (OMWW).

It can contribute to a deeper understanding of the possible biological actions of olive-related compounds and to the possibility of turning a waste product related to several environmental issues into a natural extract with a remarkable antioxidant effect, with potential applications in the veterinary, cosmetic, and pharmaceutical fields.

A maximum non-toxic OMWW extract concentration was assessed for the HepaRG cell model, based on to the cytotoxicity and genotoxicity testing results. Moreover, some concentrations showed antigenotoxic activity in co/post-exposure treatment, but this behaviour was not confirmed in the pre-exposure antigenotoxicity tests. 

This evidence lays the groundwork for future in vitro genotoxicity or antigenotoxicity assessments with different cell lines or for in vivo experiments, which are crucial and mandatory if the final aim is the development and marketing of a food supplement. Nevertheless, a baseline assessment displaying the non-toxic activity of the OMWW extract is essential, especially on the hepatic cell model since the liver is the main organ of metabolism.

## Data Availability

The data presented in this study are available on request from the corresponding author.
